# Bioinformatics approaches for deciphering the epitranscriptome: Recent progress and emerging topics

**DOI:** 10.1016/j.csbj.2020.06.010

**Published:** 2020-06-13

**Authors:** Lian Liu, Bowen Song, Jiani Ma, Yi Song, Song-Yao Zhang, Yujiao Tang, Xiangyu Wu, Zhen Wei, Kunqi Chen, Jionglong Su, Rong Rong, Zhiliang Lu, João Pedro de Magalhães, Daniel J. Rigden, Lin Zhang, Shao-Wu Zhang, Yufei Huang, Xiujuan Lei, Hui Liu, Jia Meng

**Affiliations:** a School of Computer Sciences, Shannxi Normal University, Xi’an, Shaanxi 710119, China; b Department of Biological Sciences, Xi'an Jiaotong-Liverpool University, Suzhou, Jiangsu, 215123, China; c Department of Mathematical Sciences, Xi'an Jiaotong-Liverpool University, Suzhou, Jiangsu, 215123, China; d AI University Research Centre, Xi’an Jiaotong-Liverpool University, Suzhou, Jiangsu 215123, China; e Institute of Ageing & Chronic Disease, University of Liverpool, L7 8TX, Liverpool, United Kingdom; f Institute of Integrative Biology, University of Liverpool, L69 7ZB Liverpool, United Kingdom; g School of Information and Control Engineering, China University of Mining and Technology, Xuzhou, Jiangsu 221116, China; h Key Laboratory of Information Fusion Technology of Ministry of Education, School of Automation, Northwestern Polytechnical University, Xi’an, Shaanxi 710072, China; i Department of Electrical and Computer Engineering, University of Texas at San Antonio, San Antonio, TX, 78249, USA; j Department of Epidemiology and Biostatistics, University of Texas Health Science Center at San Antonio, San Antonio, TX 78229, USA

**Keywords:** Epitranscriptome, RNA modification, Bioinformatics approaches, Recent progress, Future perspective

## Abstract

Post-transcriptional RNA modification occurs on all types of RNA and plays a vital role in regulating every aspect of RNA function. Thanks to the development of high-throughput sequencing technologies, transcriptome-wide profiling of RNA modifications has been made possible. With the accumulation of a large number of high-throughput datasets, bioinformatics approaches have become increasing critical for unraveling the epitranscriptome. We review here the recent progress in bioinformatics approaches for deciphering the epitranscriptomes, including epitranscriptome data analysis techniques, RNA modification databases, disease-association inference, general functional annotation, and studies on RNA modification site prediction. We also discuss the limitations of existing approaches and offer some future perspectives.

## 1 Background

Post transcriptional RNA modification occurs on all types of RNA and plays a vital role in regulating every stage of RNA life. More than 170 different types of RNA modifications have been identified, and the majority of them are methylation modification. Ribonucleoside modification contains many chemical components that are added to A, G, C or U. Some modifications come from non-enzymatic processes or oxidative damage. Others are RNA editing, which was originally described as a process of adding polyuridine residues to selected RNAs coding regions. Since then, editing has expanded to include the removal or addition of RNA bases, although further differences remain somewhat inconsistent. There are 111 modifications that can be found in tRNAs, 33 in rRNAs, 17 in mRNA, 11 in lncRNAs and other noncoding RNAs [1], [2].

However, due to the lack of effective means to detect RNA methylation, the research on RNA methylation had long been stagnant. For a long time, researchers had considered RNA modification as a mechanism of fine-tuning gene expression regulation, and mostly limited to noncoding tRNA and rRNA. The importance of RNA modifications was not fully aware of until the discovery of human fat mass gene FTO as a RNA m^6^A demethylase [3] and the invention of a transcriptome-wide m^6^A profiling approach MeRIP-seq (or m^6^A-seq) [4], [5]. These techniques give us a global view of mRNA modification by providing detailed maps of 12 modifications that can be incorporated into the transcriptome, including N6-methyladenosine (m^6^A), pseudouridine (ψ), N4-acetylcytidine (ac^4^C), N1-methyladenosine (m^1^A), N7-methylguanosine (m^7^G), 2′O-methylations (Cm, Am, Gm, Um), 5-methylcytidine (m^5^C), 5-hydroxymethylcytidine Cytidine (hm^5^c) and inosine (I). Please refer to two recent reviews in this perspective [1], [2].

N^6^-methyladenosine (m^6^A) is the most abundant and the most well studied chemical modification on eukaryotic mRNA [6], whose changes in level (and the consequent biological effects) are mediated by methyltransferase (writer), demethylases (eraser) and recognition proteins (reader) [7]. The methylation is catalyzed by the protein complexes of writer (including mainly METTL3, METTL14 and WTAP as well as KIAA1429, RBM15 and RBM15B) and is de-methylated by the demethylases such as ALKBH5 [8] and FTO [9]. Quite a few proteins involved in the formation of the reader complexes that specifically recognized m^6^A sites have been discovered. These include YTH family proteins (YTHDF1-3, YTHDC1) [10], [11], [12], [13], transcription initiation complex eIF3 [14], ribonucleoprotein HNRNPA2B1 [15] and HNRNPC [16]. Tens of thousands of m^6^A sites have been identified in the transcriptome, suggesting that this modification may have a wide-ranging effect on gene expression regulation [17]. For example, mediated gene silencing on the X chromosome by the long non-coding RNA X inactive specific transcripts (XIST) through YTHDC1 is due to the recognition of the m^6^A sites [18]. In addition, m^6^A can also affect translation extension by influencing the anticodon pairing rate and fidelity of both mRNA and tRNA [19]. Furthermore, m^6^A modification is also involved in the gene regulation of histone modifications [20] by recruiting CCR4-NOT complex which promoted degradation of targeted RNA after YTHDF2 binding to m^6^A site [11], [21]. The latter increases cap-independent translation under UV radiation or heat shock through the binding of the transcription initiation complex eIF3 binding to m^6^A sites in the 5′UTR region [22]. There are many reported functions of m^6^A modifications, including but not limited to, promoting the learning and memory capability of mice by m^6^A-mediated binding of the protein YTHDF1 to mRNA [23] and regulating the clearance of the mRNAs that affect embryonic development in zebrafish through YTHDC2 protein recognition of m^6^A sites on mRNAs [24]. In living organisms, enhancement of microRNA maturity can be achieved by m^6^A methylation of pri-miRNA [25]. Meanwhile, this kind of modification is also important for maintaining methyl-donor-S-adenosylmethionine (SAM) levels [26]. Inhibition of RNA methylation by reducing m^6^A demethylase can disrupt the circadian clock and extend the circadian clock cycle, while overexpression of demethylase shortens the cycle [27]. This is also supported by other studies that extensive mRNA stabilization occurs in cells, especially those mRNAs that encode proteins related to the circadian clock [28]. The m^6^A-mediated regulation of mRNA stability also plays important roles in stem cell differentiation [29], [30], [31]. Importantly, various changes in m^6^A methylation are presented in a large number of mRNAs encoded by genes associated with human diseases, especially cancers [32], suggesting that this modification could be targeted as the biomarkers for disease diagnosis or interference, for instance, in viral infection [33], cancer [34], [35], [36], acute myeloid leukemia [37], T Cell-related diseases [38] and certain brain disorders, such as autism, Alzheimer's disease and schizophrenia [39]. Inhibition of the activity of fat and obesity-related protein (FTO) by R-2-hydroxyglutaric acid (R-2HG) increases the overall m^6^A level in R-2HG sensitive leukemia cells which in turn decreases the stability of MYC/CEBPA transcripts, leading to suppression of MYC/CEBPA-related signaling pathways and thus inhibition of the proliferation of FTO-highly expressed cancer cells [40].

5-methylcytosine (m^5^C) is a wild-spread post-transcriptional RNA modification that has been detected in rRNA, tRNA, mRNA, lncRNA, viral RNA and etc. [41], [42], [43]. In mammals, m^5^C was primarily catalyzed by RNA methyltransferase DNMT2 and NSUN2 along with its homologs [44], [45], [46]. Likewise, the m^5^C marks on RNAs formed by NSUNs can contribute to promoting the overall protein synthetic level in cells in terms of protecting tRNA from shearing or promoting the formation of ribosomes [47], [48]. Another study indicated that the deficiency of NSUN2 in T cells may result in the loss of the m^5^C addition on the HIV-1 mRNA and perturb the translation of HIV-1 mRNA by inhibiting the recruitment of ribosomes and alternative splicing of viral RNA [49]. In human, YBX1 has been identified as a novel “reader” of m^5^C modification, preferentially recognizing methylation C by its cold shock domain (CSD) [50] and contributing to promote the maintenance, proliferation and differentiation of adult stem cells [51]. m^5^C deposition on RNAs will vary their stability among different RNA species. It may prevent mRNA decay though the binding of YBX1 with mRNA stabilizer PABPC1A [50] and another example proposed mRNA stability could be ensured by NSUN2 and YBX1, driving the pathogenesis of human bladder urothelial carcinoma [52]. While recently it has been reported that viral ncRNA level increased followed by the loss of m^5^C modification, which was mediated by the ablation of NSUN2 [53]. Latest studies in this year have related m^5^C modification with the regulation of the thermal adaptability in animal and plant cells by regulating the functions of tRNA and mRNA [54], [55]. A well-described hydroxymethylcytosine (hm5C) modification in DNAs was also observed in RNAs, which is oxidized form of m^5^C exhibiting important physiological roles in drosophila [56].

Adenosine-inosine (A-to-I) RNA editing is the main form of RNA editing in mammals [57], mediated by the members of the adenosine deaminase acting on RNA (ADAR) enzyme family, which will hydrolyze adenosine to inosine, first discovered in 1987 [58]. Guaranteed by the development of deep sequencing and advances in bioinformatics, 4.5 million A-to-I RNA editing sites have been confirmed by high throughput screening, either in the coding or non-coding region [59]. Due to the chemical properties of inosine are similar to guanosine, it will complement with cytidine to form base pairs [60]. This nucleotide conversion may affect gene expression, regulation and functions, in terms of the changes of amino-acid sequence, the process of miRNA expression and maturation, depending on the areas where the RNA editing events occur [61], [62]. Studies have showed that the dysregulation of A-to-I RNA editing in transcriptome or abnormal expression of ADAR is associated with various diseases, including neurological diseases, immune disease, cancer, viral infections and etc. For example, A-to-I RNA editing in a subunit of glutamate receptor 2 (GluR2) is essential for the survival of motor neurons, hence the inhibitors targeting non-A-to-I RNA editing receptors may serve as an additional tool for the treatment of amyotrophic lateral sclerosis (ALS) [63]. A-to-I RNA editing on some non-coding sites is believed to have some key roles for innate immunity [64], [65]. For instance, A-to-I RNA editing activities have a significant diversity in response to different subtypes of influenza A virus in human epithelial cells [66]. A latest example found the regulatory role of the A-to-I RNA editing in the post-transcriptional control of rheumatoid arthritis (RA), which could be used in clinical treatment as a therapeutic target [67]. During the occurrence and development of human tumors, the A-to-I RNA editing level on some oncogenes and tumor suppressor genes also been found in disorder, but either high or low is contingent on the specific cancer type [58], [68], [69]. A study indicated that inhibition of ADAR1 significantly inhibited proliferation, invasion and migration in a thyroid tumor cell model [70]. Another research proved that A-to-I RNA editing can contribute to the proteome diversity of breast cancer by the changes of amino acid sequence [71].

Our knowledge of RNA modification is continuing to expand rapidly. In addition to the widely studied m^6^A methylation, pseudouridine (ψ) is one of the most abundant [2] and extensively studied types of transcriptome modification of RNAs in living organisms. It is formed by isomerization of uracil nucleoside (U). The isomerization of uracil and pseudouracil is catalyzed by PUS enzymes alone or together with H/ACA ribonucleoproteins [72]. Ψ has a key function in guiding the process of protein translation in stem cells. It is of great potentials for the treatment of stem cell related diseases, such as human myelodysplastic syndrome [73]. Pseudouracil also reduces RNA conformational variability, enhances base pairing stability and polar interactions with proteins, and thereby regulating mRNA stability and gene expression [74]. It has been reported that pseudouracil nucleoside (ψ) is involved in heat shock response from yeast, but its molecular mechanisms remains unclear [75]. Several new types of RNA modifications have also been reported recently. For example, a well-described hydroxymethylcytosine (hm^5^C) modification in DNAs was also observed in RNAs, exhibiting important physiological roles in drosophila [56]. N^4^-acetylcytidine (ac^4^C) is revealed as a mRNA modification catalyzed by the acetyltransferase NAT10 [76]. N^6^,2′-O-dimethyladenosine (m^6^A_m_) in mRNA is another type of reversible methylation, and its modification status in the 5′ cap influences stability of cellular mRNAs [77], [78]. N^1^-methyladenosine (m^1^A) methylation is a newly discovered reversible epigenetic modification that can be de-methylated by RNA repair enzyme ALKBH1 [79], but there not have been identified yet about m^1^A methylation enzymes and its recognition proteins. Unlike m^6^A, m^1^A has even lower abundance, predominantly distributed in the 5′UTR region of mRNA which may be involved in regulating translation initiation process [80]. At last, 2′-O-methylation (Nm) of viral own genomes, such as HIV-1 and WNV RNA viruses, may help their escape from host innate immune responses [81], [82]. The above reviewing has that RNAs undergo a number of chemical modifications, which play various critical roles in physiological and pathological processes in nearly all organisms.

**Fig. 1:**
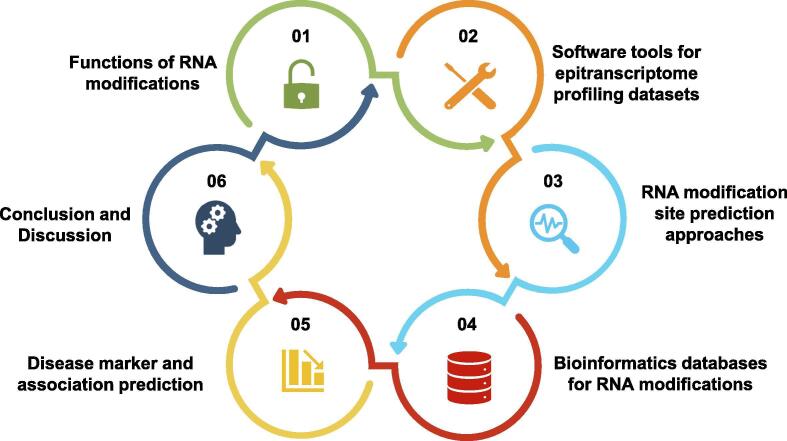
The emerging topics in epitranscriptome bioinformatics.

Thanks to the development of sequencing technologies, transcriptome-wide profiling of RNA modifications has been made possible by a number of different techniques such as MeRIP-seq and RNA BS-seq. With the accumulation of large amount of high-throughput datasets, bioinformatics approaches have been increasingly needed for unraveling the epitranscriptome as a cost effective avenue. We systematically reviewed in the following the emerging topics and recent progress in bioinformatics approaches for deciphering the epitranscriptomes, including epitranscriptome data analysis techniques, RNA modification databases, disease-association inference, general functional prediction, and RNA modification site prediction methods (especially deep learning approaches). The review is divided into the following sections. We firstly introduced the tools for epitranscriptome data analysis as well as some epitranscriptome profiling technologies. Then we summarized the algorithms for methylation sites prediction and the existing databases dedicated for RNA modifications. We in the next analyzed the disease marker and association prediction related to RNA modifications. In the end, we briefly discussed the limitations of existing epitranscriptiome bioinformatics approaches and offered some possible future perspectives (see Fig. 1).

## 2 Tools for epitranscriptome data analysis

With the advances in next-generation sequencing approaches, many experimental methods have been designed to profile various types of RNA modified nucleotides. Meanwhile, a number of computational programs were developed for the analysis of the massive high-throughput sequencing data generated. We briefly reviewed here a few software tools developed for epitranscriptome profiling data generated from MeRIP-Seq, RNA BS-Seq, etc.

### 2.1 MeRIP-Seq (or m^6^A-Seq)

**Fig. 2:**
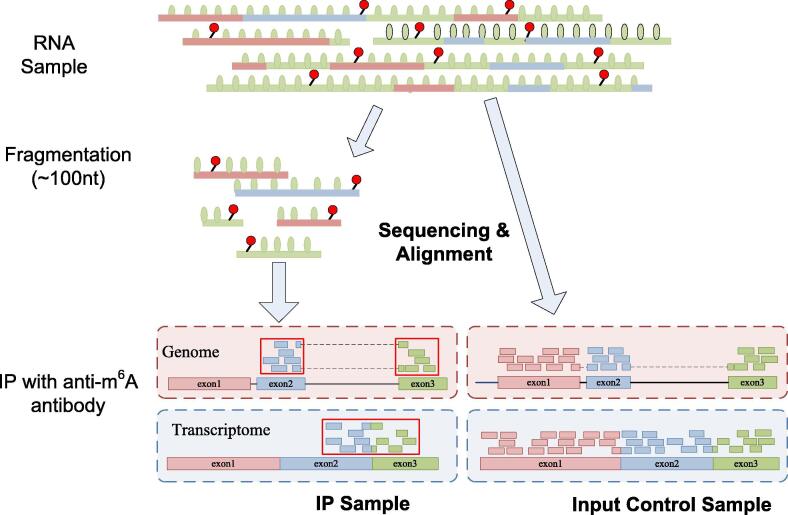
Illustration of MeRIP-Seq Protocol. In MeRIP-Seq, two types of samples (IP and control samples) are generated. In the beginning of the protocol, RNA molecules are firstly sheared into fragments of around 100 nt. Through anti-m^6^A antibody, the IP sample provides unbiased measurement of the methylated RNA fragments; the control sample reflects the basal RNA abundance and is used as a negative control, by comparing to which, the peaks (or methylated sites) can be identified. The exomePeak approach seeks to identify enriched regions on pooled exons so that a single peak may not be split into multiple enriched regions on the genome.

Methylated RNA Immunoprecipitation sequencing (MeRIP-Seq or m^6^A-Seq) [4], [5] is so far the most widely adopted experimental approach for profiling the transcriptome-wide distribution of RNA modification (see Fig. 2). Considered as a marriage of ChIP-Seq and RNA-Seq, it is possible to infer from the data generated from this antibody-based approach the location of RNA modification (peak calling) as well as the changes in methylation status between two different biological conditions (differential methylation analysis). Additionally, site clustering analysis of epitranscriptome data from multiple biological conditions may reveal co-methylation patterns, which may provide insights into the regulatory mechanisms of the epitranscriptome by relevant protein regulators (Writers, Erasers, and Readers). These perspectives are reviewed as follows:

#### 2.1.1 Peak Calling (or Site Detection)

MeRIP-Seq immunoprecipitates the RNA fragments containing the modification with anti-m^6^A polyclonal (SYSY, RN131P) or monoclonal (17-3-4-1) antibodies in randomly interrupted RNA fragment library to construct the IP sample, so the segments carrying the methylation mark are over-represented in the IP samples compared with the input control sample, which can be viewed as the standard RNA-Seq library. Mapping the reads of IP sample to the reference genome will form a peak of reads coverage near the modification sites. Through the statistical analysis of IP over input enrichment on the genomic sliding windows, we can infer the locations of m^6^A modification sites across the genome or exome. Several computational tools have been developed for site detection (peak calling) from MeRIP-seq data (see Table 1**)**, and among them, MACS [83] has been a popular peak calling tool originally developed for analyzing ChIP-Seq data, but it is also frequently used to process MeRIP-Seq data by many published studies [84]. Another widely applied software tool is exomePeak, which was specifically designed for epitranscriptome peak calling of MeRIP-Seq data [85], with a major update released recently (exomePeak2). The major advancement of exomePeak2 compared with MACS is its ability to account for the technical and biological variabilities that are predominant in RNA-Seq compared with DNA-Seq [86]. For example, exomePeak2 implements the functionality of sequence content bias correction. Consequently, it could dramatically reduce the systematic errors generated by PCR amplification during the library preparation of IP and input samples.

**Table 1:** Site prediction tools from MeRIP-seq data.

Tool	Input format	Description	URL/stand-alone package	Reference
MeRIP-PF	FASTQ	MeRIP-PF segments the reference genome by a fixed window, then compares reads count between control sample and IP sample to obtain p-value and adjusted p-value by Fisher’s exact test and Benjamini-Hochberg method. In order to form real peaks, the significant and adjacent windows are concatenated.	http://software.big.ac.cn/MeRIP-PF.html	[177]
BaySeqPeak	Reads count matrix	A Bayesian hierarchical model is proposed to detect methylation sites from MeRIP-Seq data in BaySeqPeak. By using zero expansion negative binomial model combined with hidden Markov model, the spatial dependence of enrichment of adjacent reading segments is explained, which has better stability under the condition of small samples.	https://github.com/liqiwei2000/BaySeqPeak	[178]
MACS2	BAM	Use a Poisson model to detect peaks. It was originally developed for ChIP-seq data analysis, but has also been quite popular in MeRIP-seq data analysis for its reliability, speed and convenience.	https://github.com/taoliu/MACS/	[83]
exomePeak	BAM	Exomepeak uses przyborowski and wilenski methods to compare the mean value (or C-test) of two Poisson distributions. It can detect the peak across the exon connection region of a specific gene exon set. The version 2 (exomePeak2) was released very recently.	https://rdrr.io/github/ZhenWei10/exomePeak2/	[85]
m6AViewer	BAM	m6AViewer constrains the number of read segments and the width of the peak, and uses EM algorithm to find the most possible m6A methylation peak.	http://dna2.leeds.ac.uk/m6a	[179]
MeTPeak	BAM	MeTPeak models reads count, introduces the layer with beta variable to obtain the variance, and uses the hidden Markov model to describe the reading dependency near the site.	https://github.com/compgenomics/MeTPeak	[180]

#### 2.1.2 Differential methylation analysis

**Fig. 3:**
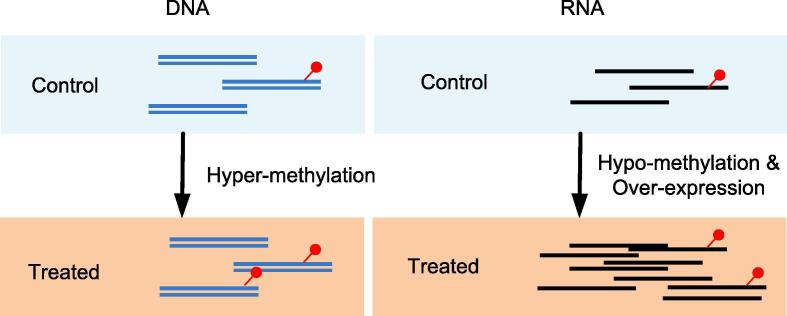
RNA methylation and DNA methylation. Compared with the control group, both the absolute and relative amount of methylated DNA in the treatment group increased under the treated condition; however, for the two may not be consistent for RNA methylation. In the above example, the total amount of methylated RNA increased in the treatment group; however, due to the increased expression level (over-expression), the relative amount of methylated RNA decreased (hypo-methylation).

Differential methylation analysis focuses on the changes of modification status between two different biological conditions. Although changes in absolute abundance of methylation may be equally important, current formulation overwhelmingly focused on the relative abundance or methylation proportion, specifically, the ratio of methylated to total RNAs, as previously modeled by [87]. Different from DNA differential methylation, for differential RNA methylation analysis, it is important to account the changes in basal expression levels of the RNA transcripts, especially for the relative abundance comparison (See Fig. 3). We summarized the existing methods for RNA differential methylation in Table 2**.** Although classic approaches based on Fisher’s exact test (realized in the exomePeak R package) [87] has been quite popular in differential methylation analysis for its modeling robustness and implementation easiness, recent published methods such as RADAR [88] and QNB [89] have achieved higher accuracy through specifying refined statistical models.

**Table 2:** Differential methylation analysis tools from MeRIP-seq data.

Tool	Input format	Description	URL/stand-alone package	Reference
exomePeak	BAM	The original exomePeak uses a rescaled version of Fisher’s exact test to detect differential methylation sites. The latest version of exomePeak2 uses a generalized linear model which considers the over-dispersion of reads count and GC content bias in sequencing data.	https://rdrr.io/github/ZhenWei10/exomePeak2/	[87]
FET-HMM	BAM	FET-HMM divides the detected RNA methylation site into small bins and uses a hidden Markov model to detect differential methylation sites.	https://github.com/lzcyzm/RHHMM	[181]
MeTDiff	BAM	MeTDiff for differential methylation sites models reads variations with beta-binomial model. Then a likelihood ratio test based on the beta-binomial is developed to test the significance of differential methylation sites.	https://github.com/compgenomics/MeTDiff	[182]
RADAR	Reads count matrix	RADAR enables accurate identification of altered methylation sites by accommodating variability of pre-immunoprecipitation expression level and post-immunoprecipitation count using different strategies.	https://github.com/scottzijiezhang/RADAR	[88]
DRME	Reads count matrix	DRME aims at RNA differential methylation in small samples. It uses two independent negative binomial distributions to model the reads count in the methylation region, and uses two-dimensional local regression to estimate variance for solving the effect of transcription regulation, and carrying out RNA differential methylation analysis combined within-group biological variability difference of biological replication samples.	https://github.com/lzcyzm/DRME	[183]
QNB	Reads count matrix	QNB is based on four independent negative binomial distributions with the variances and means for reads count of the input control samples and IP samples, and linked by local regressions. QNB combined information from both input and IP samples to estimate gene expression, which could improve the testing performance for lowly expressed genes.	https://cran.rstudio.com/web/packages/QNB/	[89]

#### 2.1.3 Clustering analysis of m^6^A sites (peaks)

**Fig. 4:**
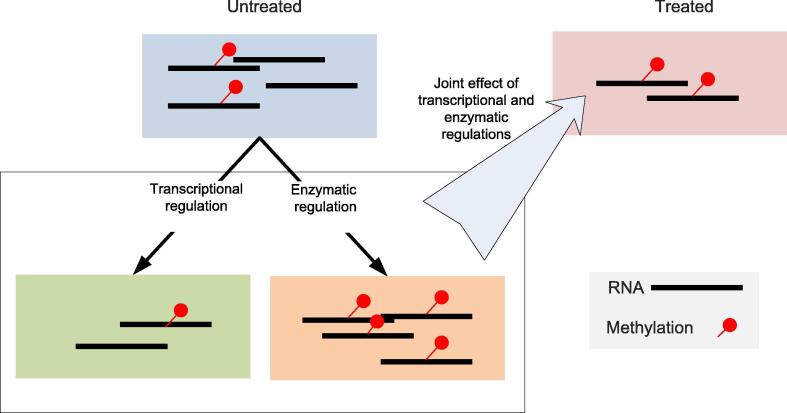
The regulation of RNA methylome. The dynamics in epitranscriptome are a result of a joint effect of both transcriptional and enzymatic regulations. On the one hand, transcriptional regulation directly changes the amount of RNA molecules and leads to coordinated changes in the absolute amount of methylated molecules, leaving the relative amount unchanged. On the other hand, enzymatic regulation of the RNA methylome by ‘methylation potential’ changes directly the percentage of methylated molecules. For the above illustration, under the joint effects of transcriptional down-regulation and enzymatic hypermethylation, the absolute amount of methylated RNA stays unchanged.

Epitranscriptome serve as an important layer of post-transcriptional regulation, and the count-based quantification in MeRIP-Seq data could potentially shed light on the mechanics of conditional specific regulation through RNA modification. Although the transcript specific regulatory mechanism of the epitranscriptome is still unclear, the clustering partition of the methylation sites on distance metric evaluated through the conditional specific methylation profiles may associate with the targeting of RNA modification regulators (Writers, Readers, Erasers) (See Fig. 4). Some relevant works in the field were summarized in Table 3. Although classical clustering algorithm such as hierarchical clustering and K-means clustering can be reasonably efficient under homogenous laboratory conditions [90], recent developments on technical independent quantification method could fundamentally improve the quality of the clustering partition after the stratification of the major technical variables from the methylation level estimates.

**Table 3:** Summary of the studies for m^6^A methylation clustering.

Method	Input format	Description	URL/stand-alone package	Reference
MeTCluster	BAM	MeTCluster, a novel algorithm and an open source R package, models the reads count variance and the underlying clusters of the methylation peaks by a hierarchical graphical model. It is evaluated on both simulated and real MeRIP-Seq datasets.	http://compgenomics.utsa.edu/metcluster	[87]
Binary Clustering	M-value	Perturbation of m^6^A writers reveals two distinct classes of mRNA methylation at internal and 5′ sites		[184]
Four clustering methods	M-value	Four different clustering approaches are used, including K-means, hierarchical clustering (HC), Bayesian factor regression model (BFRM) and nonnegative matrix factorization (NMF) to unveil the co-methylation patterns in epitranscriptome.		[185]
Threshold-Based Measurement Weighting	Reads count matrix	A convenient measurement weighting strategy that can largely tolerate the artifacts of high-throughput sequencing data.		[186]
DPBBM	Reads count matrix	DPBBM implements a beta-binomial model, which uses the original measurement value based on count instead of the estimation value to capture the clustering effect on the methylation level. In addition, the nonparametric Dirichlet process is used to automatically determine the optimal number of clusters, which avoids the common problem of model selection in cluster analysis.	https://cran.r-project.org/web/packages/DPBBM/	[187]

#### 2.1.4 Quality assessment

The quality of MeRIP-seq data can be evaluated by the conventional 2nd generation sequencing quality control pipeline such as FASTQC. Recently, trumpet R package is developed for MeRIP-Seq specific quality examination [91]. The trumpet package takes the aligned BAM files as the inputs and returns an assessment report with a single line of R command concerning the quality statistics of sequencing reads distribution, the strength of the immunoprecipitation signal, and the comparison between different biological replicates. After the systematic examination of the published MeRIP-seq experiments, we observed substantial amount of technical biases in a significant proportion of the published MeRIP-seq samples. The technically effected samples should be handled carefully through the quantification methods independent of the source of errors while performing any downstream data analysis tasks.

### 2.2 Reverse transcription signature in sequencing

The combination of Reverse Transcription (RT) and high-throughput sequencing has emerged as an effective approach for identification of RNA modification through the analysis of the misincorporation during complementary DNA (cDNA) synthesis or abortive RT-products [92]. Before the advent of direct RNA sequencing technique nanopore [93], the modified RNA templates were reversed transcribed into cDNA to generate RNA-seq data. As the newly synthesized cDNA contains only four types of canonical deoxynucleotides, this process may lead to the partially or completely loss of information about RNA modifications stored on the original RNA templates [94]. To solve this problem, some chemical reagents were used to specifically react with a target modification, which in return alter the cDNA synthesis at modified RNA sites, i.e., the use of N-cyclohexyl-N′-(2-morpholinoethyl)-carbodiimide-metho-p-toluenesulfonate (CMC) to leave a bulky group on pseudouridine modification and stop reverse transcription [74]. Besides, other RNA modifications with chemical groups added on their Watson-Crick face do not need chemical derivatization to alter cDNA synthesis. For example, the m^1^A RNA modification has a methyl group on the Watson-Crick face of adenosine, which resulted in cDNA synthesis differing from that of an unmodified adenosine on the RNA template. For the RNA templates containing m^1^A modification, the products of transcription arrest were presented in the synthesized cDNA, i.e., incorporation of mismatched dNTPs at modification position and abortive cDNA fragments, and this erroneous information generated during cDNA synthesis was termed as reverse transcription signatures. The signals of RT arrest were traditionally detected by either capillary electrophoresis or polyacrylamide gelelectrophoresis (PAGE) [95] before the development of deep sequencing methods. As the product of an A-to-I deamination, inosine is reversed transcribed for a cytidine rather than a thymidine during cDNA synthesis. And the first transcriptome-wide mapping of a RNA modification was benefited from this model misincorporation [96]. Also, combined analyses of both mismatch patterns plus defined RT arrest rate were performed to efficient identify 1-methyladenosine (m^1^A) modification [92]. The bioinformatics tools used for reverse transcription signature analysis were listed in Table 4.

**Table 4:** Summary of the reviewed tools for RT signature analysis.

Tool	Input format	Description	URL/stand-alone package	Reference
Coverage Analyzer (CAn)	SAM	CAn is a tool that offers the functions of inspection and visualization of deep sequencing data to identify RNA modification, which combines a pipeline to process data with flexible controls for differential or independent visualization and systemically screening for modification candidates using RT signatures.	https://zenodo.org/record/164811 (doi:https://doi.org//10.5281/zenodo.164811) or https://sourceforge.net/projects/coverageanalyzer/	[188]
HAMR	BAM	HAMR is a tool that allows fast identification of RNA modification at single-nucleotide resolution using the nucleotide substitutions identified from RNA-seq datasets, which scans modification candidates either transcriptome-wide or specific locations by interested genomic coordinates.	http://wanglab.pcbi.upenn.edu/hamr	[189]
Galaxy modification calling pipeline	FASTQ	A modification calling pipeline based on Galaxy, which provides a versatile graphical workflow system for modification sites calling based on machine learning. The machine learning module in downstream analysis offers quality assessment parameters to help to improve the experimental parameters for both library preparation and sequencing.	https://github.com/HelmGroup	[94]

### 2.3 RNA bisulfite sequencing

5-Methylcytosine (m^5^C) is a type of chemical modification on the carbon 5 atom of cytosine, which can be detected by high-throughput sequencing of RNA treated by bisulfite (RNA Bisulfite sequencing) that converts all unmodified cytosine into uracil leaving modified cytosine (m^5^C) unaffected [97]. Although with some intrinsic bias, it has been considered as the gold standard for profiling m^5^C epitranscriptome [98], [99]. One of the primary advantages of RNA Bisulfite sequencing in detection of modified cytosine (m^5^C) is that a transcriptome-wide view of m^5^C modification at single-based resolution can be provided. It is worth noting that, although both 5-methylcytosine and 5-hydroxymethylcytosine are resistant to deamination and thus can not be differentiated by bisulfite sequencing, the extremely low level of 5-hydroxymethylcytosine in human and mouse mRNAs [100] still makes RNA Bisulfite sequencing to become a robust and attractive technique for profiling m^5^C epitranscriptome. Several toolkits support the analysis of data generated from RNA Bisulfite sequencing (Table 5). It is important to note that a number of remedies have been taken to eliminate false positive sites reported from RNA Bisulfite sequencing technique, including statistical methods, excluding low quality reads, filtering of bisulfite conversion-resistant sites and SNPs, etc. [68], which are often necessary.

**Table 5:** Summary of the reviewed tool kits to process data generated from RNA-BisSeq technique.

Tool	Aligner	Description	Programlanguage	URL/stand-alone package	Reference
meRanTK	meRanT: Bowtie2 meRanG: STAR or TopHat2	meRanTk contains five multithreaded programs including meRanT, meRanG, meRanCall, meRanCompare, and meRanAnnotate, which is the first publicly available tool for high-throughput RNA cytosine methylation data analysis.	Perl	http://icbi.at/software/meRanTK	[104]
BS-RNA	HISAT2	BS-RNA features in its ability to map ‘dovetailing’ reads using BEERS, compared with pervious published tool meRanTK.	Perl	http://bs-rna.big.ac.cn	[190]
BisRNA	BSMAP	BisRNA provides a computational tool that features in combining data-driven statistics modeling and tailored filtering together to reduce possible artifact introduced by bisulfite sequencing.	R	https://cran.r-project.org/web/packages/BisRNA	[191]
Episo	Bowtie	Episo is the only computational tool available to quantify the RNA m^5^C modification at the transcript isoform level, which distinguish m^5^C level between isoforms of the same gene.	GNU GPLv3 + license	https://github.com/liujunfengtop/Episo	[192]

#### 2.3.1 Quality control of raw RNA Bisulfite sequencing data

In the process of sodium bisulfite interaction and cDNA conversion, unmodified cytosine in mRNA will end up as thymine, and the GC contend is extremely low in mRNA Bisulfite sequencing data. Therefore, quality control of bisulfite sequencing reads should be implemented, low quality bases and adaptor sequences should be trimmed off from the raw data. Software tool such as Trimmomatic [101] can be used in this regard.

#### 2.3.2 Alignment of RNA bisulfite sequencing reads

Reads generated from bisulfite sequencing can be mapped to either a reference transcriptome or an annotated genome, using aligners such as Bowtie2 [102] and HISAT2 [103]. For the alignment of bisulfite sequencing reads to a reference transcriptome, the longest transcript with the highest aligned score were considered, when it comes to issue that reads may be mapped to multiple transcripts of the same gene [104]. To increase the overall mapping rates of bisulfite sequencing data, sequencing reads may be aligned to an annotated genome first, and then a reference transcriptome can be used for further alignment against unmapped reads [105].

#### 2.3.3 Methylation calling and elimination of false positive sites

To avoid the detection of false positive sites, strict filters and statistical methods were applied during methylation calling process [105], [106], [107], [108], along with the selection of bisulfite conversion-resistant bases using RNA secondary structure prediction tools [107]. Besides, the m^5^C methylation candidates were further filtered to remove those sites that overlapped with known genetic variants and RNA editing sites [109], using databases such as dbSNP [110] and REDIdb [111]. Several mRNA methylation studies have applied strict filtering pipelines to reduce false positive detection. Reads filters with strict criteria were set for the first step, this helps to remove PCR duplicates [107], [108] and reads with high level of unconverted cytosine rate [105], [106]. Sites filters were then applied for coverage depth, methylation level, base quality, and false discover rate (FDR). Furthermore, other filtering criteria were also implemented by different methylation studies to further evaluate candidate methylation sites, e.g., the candidate methylation sites should be detected in at least two biological replicates, or excluding 10 and 7 bases on the 5′ end of forward and reverse reads from methylation calling, respectively [107].

### 2.4 Other tools for RNA modification analysis

Besides the toolkits used for processing high-throughput sequencing data mentioned above, a number of downstream computational methods have been developed to effectively facilitate the analysis, annotation and exploration of RNA methylation data.

An open R/Bioconductor package Guitar [112] was developed to profile the transcriptomic view of RNA-related biological features represented by genome-based coordinates, which extracts the RNA coordinates relating to the landmarks of RNA transcripts and contributes to the efficiently analysis of massive amount of RNA-related biological features. RNAModR and MetaPlotR [113] were designed with similar purpose. RNAModR serves as an R-based package for the transcriptome-wide analysis and visualization of distribution of mRNA modifications, revealing the potential insights into the biological functions of these modified nucleotides. MetaPlotR was developed as a simple pipeline to help biologists with little bioinformatics knowledge to generate metagene plots of RNA modifications, protein binding sites, etc. A web application txCoords [114] can be used for peak re-mapping, therefore corrects the wrong labeled peaks and retrieves the true sequences.

RNAmod (https://bioinformatics.sc.cn/RNAmod) [115] is an interactive, one-stop, web-based platform for the automated analysis, annotation, and visualization of mRNA modifications in 21 species with 7 kinds of RNA modifications. RNAmod firstly extracts gene features (sequence length, GC content et.al) from the reference genome annotation, and then maps the submitted modification sites onto different RNA features. It then performs various coverage calculations, metagene analysis, and annotations focusing on mRNAs. The annotations include: (1) site distribution among different gene features and gene biotypes; (2) coverage analysis among RNA features; (3) site distribution around transcription start/end sites; (4) site distribution around translation start/end sites; (5) site distribution around splicing junction sites; (6) comparison of gene characteristics between modified genes and other genes; (7) modified site heatmap around translation start/end sites and transcription start/end sites; (8) mRNA metagene analysis; (9) motif enrichment analysis; (10) functional enrichments for modified genes. Three functional modules are separately provided by RNAmod, single case, group case and gene case to facilitate users who have different analysis requirements. The single case module allows users to annotate RNA modifications for a single sample. The group case modules allow users to annotate and compare the distribution of modifications between two samples or even more groups. The gene case analysis module can be used to analyze the modification distribution in the context of specific gene.

The nucleotide enrichment scores can be calculated by ToNER [116] (Transformation of Nucleotide Enrichment Ratio) through analyzing RNA-seq data generated from enriched and unenriched control libraries, in particular when obtaining data from experimental replicates, which may be used to analyze epitranscriptome data generated from enrichment-based approaches.

Furthermore, to explore the effect of genetic variants on RNA modifications, m6ASNP [117] was designed for the identification of m^6^A-associated variants that target m^6^A modification sites. A variant was defined as m^6^A-associated variants if it can cause the alteration of methylation status of a m^6^A site (gain or loss), and the potential impact of m^6^A modification on diseases was revealed by incorporating the information of disease-associated SNPs derived from different databases, including GWAS catalog [118], Johnson and O’Donnel [119] and ClinVar [120]. Lastly, an all-in-one toolkit RNA Framework [121] was recently developed, which is characterized by comprehensive analysis of most HGS-based RNA structure probing and post-transcriptional modification mapping experiments. To sum up, with the advances in high-throughput sequencing techniques and the increasing interest in RNA epitranscriptome, a variety types of upstream and downstream computational tools have been developed, to best share, annotate, analyze, and take advantage of the massive amount of NGS data generated.

## 3 RNA modification site prediction

Although is being reviewed lastly in this manuscript, computational prediction of RNA modification sites actually embodied the largest number of bioinformatics studies concerning epitranscriptome bioinformatics. At present, most of the computational prediction methods relied on gold standard datasets obtained from base-resolution epitranscriptome profiling approaches, extracted predictive features and utilized machine learning or deep learning classifiers to predict putative RNA modification sites. Among all the RNA modification types, m^6^A RNA methylation is the most widely studied, and it is also the predictive target of the earliest as well as the most sophisticated predictive approaches. We summarized in Table 6, Table 7 the prediction tools for m^6^A and other types of RNA modifications, respectively. These works together greatly improved our understanding of the distribution of multiple types of RNA modifications in different species (Please see a comprehensive review [122]). It may be worth noting that, according to a recent review [122], the WHISTLE approach, which was based on SVM algorithm and 35 additional genomic features as well conventional sequence features [123], achieved so far the best performance in m^6^A sites prediction, suggesting the value and importance of increased volume of high quality training data. When restricted to methods based on DNA/RNA sequences only, the deep learning-based method DeepPromise [122] achieved so far the best prediction performance.

**Table 6:** Summary of m^6^A site prediction tools.

Tool	Method	Encoding scheme	Species	URL/stand-alone package	Reference
iRNA-PseColl	SVM	NCP; ANF	Human	http://lin.uestc.edu.cn/server/iRNA-PseColl	[193]
WHISTLE	SVM	NCP; ANF; Genome features	Human	http://whistle-epitranscriptome.com	[123]
HMpre	XGBoost	Binary; CPD; k-mer; Site Location Related Features; Features Related to Entropy; SNP Features	Human	https://github.com/Zhixun-Zhao/HMpre	[194]
iRNA-Methyl	SVM	PseDNC	Yeast	http://lin.uestc.edu.cn/server/iRNA- Methyl	[195]
pRNAm-PC	SVM	PseDNC	Yeast	http://www.jcibioinfo.cn/pRNAm-PC	[196]
RAM-ESVM	SVM	PseDNC	Yeast	http://server.malab.cn/RAM-ESVM/	[197]
m6Apred	SVM	NCP; ANF	Yeast	http://lin.uestc.edu.cn/server/m6Apred.php	[198]
RNAMethylPred	SVM	BPB; DNC; KNN score	Yeast	MATLAB package	[199]
TargetM6A	SVM	PSNP; PSDP; NC	Yeast	http://csbio.njust.edu.cn/bioinf/TargetM6A	[200]
iRNA(m6A)-PseDNC	SVM	PseDNC	Yeast	http://lin-group.cn/server/iRNA(m6A)-PseDNC.php	[201]
M6APred-EL	Ensemble	PS(k-mer)NP; PCPs; RFHC-GACs	Yeast	http://server.malab.cn/M6APred-EL/	[202]
DeepM6APred	SVM	Deep features; NPPS	Yeast	http://server.malab.cn/DeepM6APred	[203]
iMethyl-STTNC	SVM	PseDNC; PseTNC; STNC; STTNC	Yeast	No	[204]
PXGB	XGBoost + PSO	PSNP; PSDP; NC	Yeast	No	[205]
M6APred-EL	Ensemble	PS(k-mer)NP; PCPs; RFHC-GACs	Yeast	http://server.malab.cn/M6APred-EL/	[202]
Zhuang, Y., et al.	SVM + RF + LR	Compositional features; Position-specific features; Motif; Physiochemical features	Yeast	No	[206]
M6ATH	SVM	NCP; ANF	Arabidopsis	http://lin.uestc.edu.cn/server/M6ATH	[207]
AthMethPre	SVM	k-mer	Arabidopsis	http://bioinfo.tsinghua.edu.cn/AthMethPre/index.html	[208]
RFAthM6A	RF	PSNPF; PSDPF; KSNPF; KNF	Arabidopsis	https://github.com/nongdaxiaofeng/RFAthM6A	[209]
Zhang, J., et al.	SVM	NCP; ANF	E. coli	No	[210]
MethyRNA	SVM	NCP; ANF	Human; Mouse	http://lin.uestc.edu.cn/server/methyrna.	[211]
RNAMethPre	SVM	Binary; k-mer; MFE	Human; Mouse	http://bioinfo.tsinghua.edu.cn/RNAMethPre/index.html	[212]
SRAMP	RF	Binary; KNN; spectrum	Human; Mouse	http://www.cuilab.cn/sramp/	[213]
Gene2vec	CNN	One-hot; Neighboring state; Word embedding; Gene2vec	Human; Mouse	http://server.malab.cn/Gene2vec/	[124]
DeepM6ASeq	CNN + BLSTM	Binary	Human; Mouse	https://github.com/rreybeyb/DeepM6ASeq	[129]
iRNA-3typeA	SVM	NCP; ANF	Human; Mouse	http://lin-group.cn/server/iRNA-3typeA/	[214]
Gene2vec	CNN	One-hot; Neighboring state; Word embedding; Gene2vec	Human; Mouse	http://server.malab.cn/Gene2vec/	[124]
iRNA-3typeA	SVM	NCP; ANF	Human; Mouse	http://lin-group.cn/server/iRNA-3typeA/	[214]
Dao, F.-Y., et al.	SVM	physical–chemical property matrix; Binary;NCP	Human; Mouse	http://lin-group.cn/server/iRNAm6A/service.html	[215]
iN6-Methyl	CNN	Word2vec	Human; Mouse; Yeast	https://home.jbnu.ac.kr/NSCL/iN6-Methyl.htm	[125]
RAM-NPPS	SVM	NPPS	Human; Yeast; Arabidopsis	http://server.malab.cn/RAM-NPPS/	[216]
SICM6A	GRU	3-mer	Mouse; Yeast; Arabidopsis	https://github.com/lwzyb/SICM6A	[217]
M6AMRFS	XGBoost	Dinucleotide Binary; Local Position-Specific Dinucleotide Frequency	Human; Mouse; Yeast; Arabidopsis	http://server.malab.cn/M6AMRFS/	[218]
BERMP	RF + BGRU	ENAC; Word embedding	Human; Mouse; Yeast; Arabidopsis	http://www.bioinfogo.org/bermp	[127]

Note: PseDNC (pseudo dinucleotide composition), ANF (accumulated nucleotide frequency), NCP (nucleotide chemical property), BPB (bi-profile bayes), DNC (dinucleotide composition), NC (nucleotide composition), PSNP (positionspecific nucleotide propensity), PSDP (position-specific dinucleotide propensity), NPPS (nucleotide pair position specificity), STTNC (split-tetra-nucleotide composition), PSNSP (position-specific nucleotide sequence profile), PSDSP (position-specific dinucleotide sequence profile), MFE (minimum free energy), PCPs (physical–chemical properties), KSNPF (K-spaced nucleotide pair frequencies), KNF(K-nucleotide frequencies), CPD (chemical property with density), ENAC (Enhanced nucleic acid composition), HPCR (heuristic nucleotide physicochemical property reduction), TNC (tri-nucleotide composition), TetraNC (tetra-nucleotide composition), mRMR (Minimum-redundancy and maximum-relevance).

**Table 7:** Summary of prediction tools for non-m^6^A RNA modifications.

Type	Tool	Method	Encoding scheme	Species	URL/stand-alone package	Ref
m^5^C	Feng, P., et al.	SVM	PseDNC	Human	No	[219]
	iRNAm5C-PseDNC	RF	PseDNC	Human	http://www.jci-bioinfo.cn/iRNAm5C-PseDNC	[220]
	iRNA-PseColl	SVM	NCP; ANF	Human	http://lin.uestc.edu.cn/server/iRNA-PseColl	[193]
	M5C-HPCR	SVM	HPCR	Human	http://cslab.just.edu.cn:8080/M5C-HPCR/	[221]
	pM 5 CS-Comp-mRMR	SVM	DNC; TNC; TetraNC; mRMR	Human	No	[222]
	RNAm5CPred	SVM	KNFs; KSNPFs; PseDNC	Human	http://zhulab.ahu.edu.cn/RNAm5CPred/	[223]
	iRNA-PseTNC	SVM	PseDNC; seTNC; PseTetraNC	Human	No	[224]
	iRNA-m5C_NB	NB;RF;SVM;AdaBoost	BPB;k-mer;ENAC;XXKGAP;EIIP;PseEIIP	Human	No	[225]
	PEA-m5C	RF	Binary; k-mer; PseDNC	Arabidopsis	https://github.com/cma2015/PEA-m5C	[226]
	RNAm5Cfinder	RF	One-hot; NCP; ANF	Human; Mouse	http://www.rnanut.net/rnam5cfinder	[227]
	RNAm5Cfinder	RF	One-hot; NCP; ANF	Human; Mouse	http://www.rnanut.net/rnam5cfinder	[227]
ψ	PPUS	SVM	binary	Human	http://lyh.pkmu.cn/ppus/	[228]
	PIANO	SVM	NCP; ANF; Genome features	Human	http://piano.rnamd.com	[229]
	iPseU-NCP	RF	NCP	Human; Yeast	https://github.com/ngphubinh/iPseU-NCP	[132]
	iRNA-PseU	SVM	NCP; ANF; PseKNC	Human; Mouse; Yeast	http://lin.uestc.edu.cn/server/iRNA-PseU	[230]
	PseUI	SVM	NC; DC; PseDNC; PSNP; PSDP	Human; Mouse; Yeast	http://zhulab.ahu.edu.cn/PseUI	[231]
	XG-PseU	XGBoost	NC; DNC; TNC; NCP; One-hot	Human; Mouse; Yeast	http://www.bioml.cn/	[232]
	DeepMRMP	BGRU	One-hot	Human; Mouse; Yeast	No	[128]
	CNNPSP	CNN	DNC; NCP; ANF	Human; Mouse; Yeast	No	[130]
	iPseU-CNN	CNN	n-gram and multivariate mutual information (MMI)	Human; Mouse; Yeast	No	[233]
	EnsemPseU	SVM; XGBoost; NB; KNN; RF	k-mer; Binary; ENAC; NCP; ND	Human; Mouse; Yeast	https://github.com/biyue1026/EnsemPseU	[234]
Nm	iRNA-2methyl	Ensemble; RF	Pse-in-One	Human	http://www.jci-bioinfo.cn/iRNA-2methyl	[235]
	iRNA-PseKNC	CNN	One-hot	Human	No	[131]
	iRNA-2OM	SVM	NCP; ANF; Type 2 PseKNC	Human	http://lin-group.cn/server/iRNA-2OM	[236]
	Chen, W., et al.	SVM	NCP; ANF	Human; Mouse; Yeast	No	[237]
m^1^A	iRNA-PseColl	SVM	NCP; ANF	Human	http://lin.uestc.edu.cn/server/iRNA-PseColl	[193]
	ISGm1A	RF	NCP; ANF; Genome features	Human	https://github.com/lianliu09/m1a_prediction.git.	[238]
	iRNA-3typeA	SVM	NCP; ANF	Human; Mouse	http://lin-group.cn/server/iRNA-3typeA/	[214]
	RAMPred	SVM	NCP; ANF	Human; Mouse; Yeast	http://lin.uestc.edu.cn/server/RAMPred	[239]
A to I	iRNA-AI	SVM	NCP; ANF	Human	http://lin.uestc.edu.cn/server/iRNA-AI/	[240]
	EPAI-NC	LD-SVM	l-mers; n-gapped l-mers	Fly	http://epai-nc.info/	[241]
	PAI	SVM	PseDNC	Fly	http://lin.uestc.edu.cn/server/PAI	[242]
	iRNA-3typeA	SVM	NCP; ANF	Human; Mouse	http://lin-group.cn/server/iRNA-3typeA/	[214]
m^2^G	iRNA-m2G	SVM	NCP; ANF	Human; Mouse; Yeast	No	[243]
m^7^G	iRNA-m7G	SVM	NCP; ANF; PseDNC; SSC	Human	http://lin-group.cn/server/iRNA-m7G/	[244]
	m7GFinder	SVM	NCP; ANF	Human	www.xjtlu.edu.cn/biologicalsciences/m7ghub	[245]
D	iRNAD	SVM	NCP; ANF	Human; Mouse; Yeast	http://lin-group.cn/server/iRNAD	[246]
5hmC	iRNA5hmC	SVM	k-mer; Binary	Fly	http://server.malab.cn/iRNA5hmC	[247]
ac4C	PACES	RF	One-hot;PSNSP;PSDSP;KNF;KSNPF;PseKNC	Human	http://www.rnanut.net/paces/	[248]

### 3.1 Deep learning in RNA modification sites prediction

One prominent trend is that, deep learning-based predictive approaches seem to be able to offer better overall prediction performance compared with classic machine learning methods. In contrast of traditional machine learning methods, deep learning (DL) model can automatically extract the non-linear features. Several deep learning-based methods have been developed. These methods firstly extracted the positive modification sites from existing studies and database like Met-DB and RMBase, and then selected negative samples to build a standard data set to train and test the proposed approach.

The most widely used deep learning models are convolutional neural network (CNN), which can effectively learn the motif related features from RNA sequence, and recurrent neural network (RNN), which can learn the non-linear sequential features from RNA sequence, including long short-term memory unit (LSTM) and gated recurrent unit (GRU). Gene2vec [124], DeepPromise [122], iN6-Methyl (5-step) [125] and Deep-m6A [126] built CNN models to predict m^6^A or m^1^A modifications; BERMP [127] employed a bidirectional Gated Recurrent Unit (BGRU) model to predict m^6^A; DeepMRMP [128] adopted bidirectional Gated Recurrent Unit (BGRU) and transfer learning to predict m^6^A, m^1^A, pseudouridine and m^5^C; the DL models of DeepM6ASeq [129] consists of two layers of CNN, one bidirectional long short-term memory (BLSTM) layer and one fully connected (FC) layer.

Although most of these approaches focused on m^6^A RNA methylation prediction, it is worth noting that deep learning has also been applied to other modifications as well. For example, CNNPSP [130] and iRNA-PseKNC [131] employed convolutional neural network (CNN) to predict pseudouridine and 2′-O-methylation, respectively. And DeepPromise [122] is also able to predict m^1^A sites. Please refer to Table 7 for a summary of prediction approaches for non-m^6^A modifications.

The input features of most DL-based methods are RNA or DNA sequence except Deep-m^6^A which embedded the MeRIP-Seq reads count with RNA sequence to predict condition-specific (e.g. disease or normal) m^6^A sites. There are diverse strategies to encode the input features. We summarize in the following 4 major kinds of encoding strategies, including:

(1) one-hot embedding;
(2) RNA/DNA nucleotides to vector embedding;
(3) RNA sequence and MeRIP-Seq reads count embedding;
(4) neighboring methylation state embedding.

One-hot encoding is widely used for sequence analysis [132], [133], which encodes the ‘A’, ‘U’ (or ‘T’), ‘C’ and ‘G’ to 4-dimensional binary vectors. DeepM6ASeq, Gene2vec, DeepPromise, DeepMRMP and Deep-m6A employed one-hot encoding as part of their feature encoding strategies. RNA/DNA nucleotides to vector embedding treated one or several nucleotides as words and the whole sequence as a sentence, then transfer nucleotides to numeric vectors based on semantic analysis. BERMP treated each nucleotide as a word and trained an embedding layer together with the BGRU model to convert the input nucleotide to vector; Gene2vec regarded 3 RNA nucleotides as an RNA word and developed a neural‐network-based model to generate a 100‐dimensional feature vector for each ‘word’ and also employed RNA word embedding which treated 3 RNA nucleotides as a word, then built a dictionary to embed the whole sentence and finally trained an embedding layer to transform each integral sequence into a data table; DeepPromise adopted the enhanced nucleic acid composition (ENAC) embedding, which can simultaneously depict the nucleotides’ composition and position information based on a length-fixed window slide on RNA sequence from 5′ to 3′ termini and also RNA-embedding which took 5 nucleotides as a word and adopted similar scheme with RNA word embedding adopted by Gene2vec to transform each integral sequence into a data table; iN6-Methyl (5-step) treated 3 nucleotides as a word and employed word2vec [134] to convert the ‘word’ to a 100-dimensional vector, similar with Gene2vec. Neighboring methylation state embedding is only adopted by Gene2vec, which embedded the 250 upstream and 250 downstream candidate m^6^A sites around the predicted m^6^A site to 501-dimensional binary vectors. RNA sequence and MeRIP-Seq reads count embedding is only adopted by Deep-m6A, which firstly encoded the RNA sequence using one-hot encoding and then embedded the normalized MeRIP-Seq IP sample reads count mapped to the corresponding nucleotide’s genome position to the binary vector of each nucleotide. The embedded MeRIP-Seq IP reads count can represent the m^6^A methylation level under specific condition like cancer, which provides the power to predict condition-specific m^6^A sites.

## 4 RNA modification databases

Knowledge bases with the comprehensive collection and curation of various information related to transcriptome-wide RNA modifications are often critical for elucidating their biological functions as well as for developing bioinformatics tools. A number of works have been accomplished addressing various aspects of RNA modifications including basic properties, pathway, distribution, disease association, visualization and GO functions. We review in the following a few databases related to RNA modifications.

### 4.1 RNAMDB

RNAMDB [135], [136] contains 109 RNA modifications with basic description of the RNA modification (chemical structure of the nucleoside, common chemical name, symbol, elemental composition and mass), type(s) of RNA in which the nucleoside occurs (tRNA, rRNA, mRNA, snRNA etc.), phylogenetic occurrence of the nucleoside (archaea, bacteria, eukarya and the corresponding literature citations for each, chemical abstracts registry numbers and chemical abstracts index name), literature citation to structure assignment of the nucleoside and literature citation to the first reported chemical synthesis of the nucleoside. Moreover, other informatics resources for RNA science are available in RNAMDB including different repositories of experimental protocols which comprise established procedures that are common practices in a typical RNA lab, related link database and sister database. The latest version was updated in 2011 which is freely accessible at https://mods.rna.albany.edu/.

### 4.2 MODOMICS

The MODOMICS database [137], [138] is currently the most comprehensive RNA modification pathway source. It displays the reactions linking a modified nucleoside to its precursor(s) and to hypermodificatons. Additionally, a typical entry of a modified ribonucleoside contains information about its fundamental chemical properties, chemical structure including the standard bases (A, U, C and G) they come from and the chemical groups they contain. MODOMICS also provides many other aspects to interpret and display the information above. From the aspect of RNA sequence, MODOMICS provides a collection of modified RNA sequences of different types. Sequences are visualized with all modifications highlighted and linked to the corresponding modification records. From the aspect of proteins, the MODOMICS database currently contains information above 340 functionally characterized proteins involved in RNA modification, both functional enzymes and protein co-factors necessary for multi-protein enzymes activities. MODOMICS also adds a catalogue of ‘building blocks’ for the chemical synthesis of naturally occurring modified nucleosides. The compilation is intended to facilitate solid phase synthesis of modified RNA, and thus to foster biophysical and biochemical studies. The database is freely accessible from: https://iimcb.genesilico.pl/modomics/.

### 4.3 MeT-DB

MeT-DB (MethyTranscriptome DataBase) [139] is the first comprehensive resource for m^6^A transcriptome methylation. The MeT-DB database includes three parts, Core DB, TREW DB and Functional DB. The Core database contains context-specific m^6^A peaks and single-base sites. For each predicted m^6^A peak, its chromosomal location, including start/end position, strand information, p-value, fold enrichment and q-value were reported. Moreover, m^6^A peaks, single-based m^6^A sites, motif, peak distribution plot, gene expression profiles were available and can be downloaded from the web page. The TREW database annotates target sites of m^6^A readers, writers and erasers, then the target site was further annotated with transcript regions (5′ UTR, CDS, 3′UTR, stop codon, transcription start sites and miRNA target site) and RNA type. In addition, some useful tools are provided in the MeT-DB web interface: the table view to facilitate researchers to explore and search the data in details, the genome browser to help the user visualize and compare m^6^A peaks and functions data, and the tool module that includes Guitar Plot and m6A-Driver for investigating the functions of m^6^A methyl transcriptome. It has undergone two versions [139], [140] and is currently available at http://compgenomics.utsa.edu/MeTDB/.

### 4.4 RMBase

RMBase [141], [142] currently has the most comprehensive collection of RNA modification sites, and is aimed to decode the map of RNA modifications from epitranscriptome sequencing data. RMBase v2.0 was expanded with 566 datasets and 1,397,244 modification sites from 47 studies among 13 species. To study the distribution of RNA modifications on the transcript products, RMBase mapped their sites onto the genomic coordinates of the genes with annotation including gene types and regions. RMBase also studied the relationships between RNA modification and post-transcriptome regulation, such as, miRNA binding sites, SNPs and RBPs. Besides, RMBase also annotated the RBPs as readers, writers and erasers. All SNPs and SNVs were intersected with the RNA modification regions to identify the SNPs and SNVs that might interact with the RNA modifications. Visualized logos of modification motifs and metagenes of RNA modification plotting along a transcript model are also available. The RMBase database is freely accessible at: http://rna.sysu.edu.cn/rmbase/.

### 4.5 m6AVar

m6AVar [143] is dedicated to the investigation of the functional association between genetic variants and m^6^A modification. Raw data resource of m6Avar can be mainly categorized into two parts, SNPs and m^6^A sites. In terms of SNPs, germline and somatic variants were obtained from dbSNP and TCGA database. A large number of disease-associated SNPs were obtained from GWAS, ClinVar etc. Furthermore, all of the SNPs were annotated by gene conservation scores and deleterious levels scoring from 0 to 5. In terms of m^6^A sites, they were acquired according to different confidence levels from high to low by using various strategies. The m^6^A sites with a high confidence level were derived from 7 miCLIP experiments and 2 PA-m6A-seq experiments. The m^6^A sites that have a medium confidence level were derived from 244 MeRIP-seq experiments and m^6^A sites that have a low confidence level were derived from a transcriptome-wide prediction based on Random Forest algorithm. Furthermore, the location of each m^6^A site was annotated by the transcript structure, including the CDS, 3′UTR, 5′UTR, start codon and stop codon. Combined with SNPs and m^6^A sites, m6AVar explored m^6^A-associated variants which were defined by evaluating whether it has the potential to alter the DRACH motif or other sequence features essential for m^6^A modification. Particularly, more than 2000 disease-related variants have been identified by linking the m^6^A-associated variants with GWAS and ClinVar data. m^6^AVar also provided an user-friendly web interface with multiple statistical diagrams and genome browser through which users can browse all of the m^6^A-associated variants and search data by various criteria. The m^6^AVar database is freely accessible at: http://m6avar.renlab.org/.

### 4.6 REPIC

REPIC [144] is a newly developed database dedicated to provide a new resource to investigate potential functions and mechanisms of m^6^A modifications across 11 species. To offer insights into the cell line- or tissue-specificity of m^6^A modification, REPIC supports query of m^6^A modifications by cell lines and tissue types. Peak annotation and sample annotation are available. Peak annotation includes genomic position, fold enrichment and genomic feature. Sample annotation includes the data source, read mapping statistics, metagene profiles and results from motif analysis. To better display multiple dimensional m^6^A modification information across the entire genome, REPIC provides a genome browser to visualize m^6^A peaks, fold enrichment and gene expression. REPIC is accessible at https://epicmod.uchicago.edu/repic/index.php.

### 4.7 RADAR

RADAR [145] is a rigorously annotated database of A-to-I RNA editing. It includes a comprehensive collection of A-to-I RNA editing sites identified in humans (Homo sapiens), mice (Mus musculus) and flies (Drosophila melanogaster), which contains 1 379 403 human, 8108 mouse and 2698 fly A-to-I RNA editing sites separately. Specifically, for each editing site, annotations are curated manually, which consist of the genome, strand, associated gene, functional region within the gene (coding sequence, untranslated region, intron), associated repetitive element (Alu, repetitive non-Alu, nonrepetitive), conservation of editing to other species and the reference study in which the site was first identified. In addition, for each editing site, RADAR also includes a catalog of tissue-specific editing levels from published RNA-seq datasets. RADAR allows the search for A-to-I RNA editing sites by using any combination of the abovementioned annotations. To facilitate more detailed searches, the UCSC genome browser is used to display the overlapping gene annotations, genomic nucleotide conservation, overlapping SNP database entries and overlapping repetitive elements. The RADAR is freely accessible at http://RNAedit.com.

## 5 Disease marker and association prediction

Recent studies demonstrated that aberrant m^6^A modifications is linked to a number of pathophysiological disorders, including obesity related traits [146], [147], [148], [149], diabetes [150], aberrant germ cell formation [151], circadian period elongation [27], developmental retardation [152]. Emerging evidence suggests that m^6^A modification is involved in multiple forms of human cancer and plays a crucial role in different cancer contexts, such as in breast cancer [153], [154], acute myeloid leukemia (AML) [155], [156], [157], [158], [159], glioblastoma [160], [161], [162], lung cancer [163] and liver cancer [164]. Precise identification of disease-associated m^6^A modification can be critical for understanding the disease pathogenesis. While wet lab experiments were often restricted by their costs in time and labor, computational approaches offered a viable avenue. We briefly summarized in the following some recent works related to *in silico* identification and prediction of disease association of RNA modifications including the relevant enzymes and the sites.

RNAMethyPro [165] used a biologically conserved signature of m^6^A regulators for prediction of survivals at pan-cancer level, which was based on 25 publically available datasets encompassing 13 cancer types. However, the construction of RNAMethyPro is based on silico analysis, which is controversial for obtaining the biological and clinical characteristics related to m^6^A, as well as for determining the specific functional modules of patients at high risk. Therefore, further mechanism and independent clinical verification are still appreciated to further validate that RNAMethyPro as a robust predictive signal in a variety of human cancers.

In a study led by Li et al. [166], the molecular alterations and clinical relevance of m^6^A regulators were analyzed across more than 10,000 subjects representing 33 cancer types, and revealed significant correlation between activities of cancer hallmark-related pathways and expression levels of m^6^A regulators. Besides, the authors revealed that m^6^A reader IGF2BP3 maybe a potential oncogene for Clear cell renal cell carcinoma (ccRCC), even though they cannot reliably predict the prognosis of ccRCC patients based on the risk score according to the mRNA expression of m^6^A regulatory genes.

The m^6^AVar database [167] established the association between individual m^6^A site and various diseases via disease-associated genetic mutations that may also lead to changes of RNA methylation status. To our knowledge, this is the first large-scale prediction study that linked individual RNA methylation sites to various diseases. In addition, it is a comprehensive database, which contains m^6^A related variables that may affect the m^6^A modification, which will help to interpret variables through the m^6^A function.

In the CVm6A database [168], 190,050 and 150,900 m^6^A sites were identified in cancer and non-cancer cells, which may demonstrate putative associations to cancer pathology. But due to the limitation of m^6^A sequence dataset, CVm6A, as well as most other databases, cannot fully determine the distribution of m^6^A on lncRNAs and other non-polyA RNAs.

Based on a random walk with restart approach, DRUM [169] successfully associated individual m^6^A sites to various diseases via a multi-layered heterogeneous network consisting of m^6^Asites, genes and diseases. The genes and sites were linked by association of expression levels and methylation levels, while genes and diseases are associated according to existing gene-disease association database.

By taking advantage of the guilt-by-association principle, m6Acomet [170] can infer putative GO functions of individual m^6^A sites from a RNA co-methylation network derived epitranscriptome profiling data using hub-based or module-based methods. This is the first study for large-scale prediction of GO functions for individual m^6^A sites. However, the two methods used in m6Acomet achieved only marginal improvement compared with random guesses. Furthermore, there are more data sources, which can be integrated with RNA comethylation network to obtain more accurate functional labeling.

Very recently, An et al developed a computational approach to systematically identify cell-specific trans regulators of m^6^A through integrating gene expressions, binding targets and binding motifs of large number of RNA binding proteins (RBPs) with a co-methylation network constructed using large-scale m^6^A methylomes across diverse cell states [171]. This study provides a new perspective for the regulation of m^6^A epitranscriptome.

## 6 Summary and outlook

With an increasing number of studies revealing the essence and importance of RNA modifications in general gene expression regulation and disease pathogenesis, RNA epigenetics [172] (or epitranscriptomics [173]) has captured growing attention. Bioinformatics capacity to analyze, digest, collect and share the rapidly growing epitranscriptome profiling data is sorely needed. We reviewed recent progress and emerging bioinformatics topics concerning RNA modifications, including epitranscriptome data analysis techniques, RNA modification databases, disease-association inference, functional annotation and RNA modification site prediction. Taken together, bioinformatics developments have greatly facilitated research in the area and have enhanced understanding of the biological meaning of RNA modifications.

Nevertheless, despite the rapid progress in epitranscriptome bioinformatics, there are still a number of limitations or open questions.

First, technological bias and limitations may not have received sufficient attention during development of bioinformatics tools. For example, most of the existing RNA bisulfite data interpretation tools failed to consider the abundant RNA secondary structures that may generate a large number of false positive errors [174]. Although it has been reported that there are major discrepancies between the results of different RNA modification profiling techniques (such as in m^5^C [98], [99]), few existing site prediction approaches have carefully considered it. Furthermore, most existing site prediction tools overlooked the bias induced by polyA selection during RNA-seq library preparation, which leads to under-representation of intronic and lncRNA sites.

Secondly, although existing studies suggested that RNA modification can affect the structure of RNA [175], it is not yet clear how it affects the 3D structure of RNA molecules in general [176]. It seems likely that many RNA modifications will exert at least some of their myriad functions through affecting structures so that methods considering and predicting this consequence of modification would be highly valuable.

Thirdly, some bioinformatics pipelines have not been extended to keep up with the emergence of novel modifications arising from the development of new technologies. For example, the site prediction and disease association frameworks developed for well-studied modifications (such as the WHISTLE [123] and m6AVar [167] frameworks for m^6^A modification site prediction and disease association) have not been extended to other relatively less studied RNA modifications (such as m^1^A and Nm), even though the extension should be fairly straightforward from a computational perspective. Such basic bioinformatics infrastructure is essential and should be established for all types of RNA modifications that can be profiled transcriptome-wide at base-resolution.

## 7 Author’s contribution

Jia Meng, Hui Liu and Xiujuan Lei initialized and coordinated the project. Yi Song, Rong Rong and Zhiliang Lu reviewed the biological background of RNA modifications; Lian Liu, Song-Yao Zhang, Kunqi Chen, Shao-Wu Zhang and Xiujuan Lei summarized sites prediction approaches; Lian Liu, Zhen Wei and Shao-Wu Zhang reviewed m^6^A-seq analysis approaches; Yujiao Tang, Xiangyu Wu, João Pedro de Magalhães and Daniel J. Rigden reviewed disease association and functional prediction; Bowen Song reviewed other bioinformatics tools for epitranscriptome data analysis; Jiani Ma, Hui Liu and Lin Zhang reviewed existing bioinformatics databases. All authors read, critically revised and approved the final manuscript.

## Funding

This work has been supported by National Natural Science Foundation of China [61902230, 61972451, 31671373]; China Postdoctoral Science Foundation [2018 M640949]; Fundamental Research Funds for the Central Universities [GK201903083, GK201901010]; XJTLU Key Program Special Fund [KSF-T-01].

## CRediT authorship contribution statement

**Lian Liu:** Writing - original draft, Methodology, Investigation. **Bowen Song:** Writing - original draft, Methodology, Investigation. **Jiani Ma:** Writing - original draft, Methodology, Investigation. **Yi Song:** Writing - original draft, Methodology, Investigation. **Song-Yao Zhang:** Writing - original draft, Methodology, Investigation. **Yujiao Tang:** Writing - original draft, Methodology, Investigation. **Xiangyu Wu:** Writing - original draft, Methodology, Investigation. **Zhen Wei:** Writing - review & editing. **Kunqi Chen:** Writing - review & editing. **Jionglong Su:** Writing - review & editing, Resources, Supervision. **Rong Rong:** Writing - review & editing, Resources, Supervision. **Zhiliang Lu:** Writing - review & editing, Resources, Supervision. **João Pedro de Magalhães:** Writing - review & editing, Resources, Supervision. **Daniel J. Rigden:** Writing - review & editing, Resources, Supervision. **Lin Zhang:** Writing - review & editing, Resources, Supervision. **Shao-Wu Zhang:** Writing - review & editing, Resources, Supervision. **Yufei Huang:** Writing - review & editing, Resources, Supervision. **Xiujuan Lei:** Writing - review & editing, Resources, Supervision, Funding acquisition, Project administration. **Hui Liu:** Writing - review & editing, Resources, Supervision, Funding acquisition, Project administration. **Jia Meng:** Writing - review & editing, Resources, Supervision, Funding acquisition, Project administration.

## Declaration of Competing Interest

The authors declare that they have no known competing financial interests or personal relationships that could have appeared to influence the work reported in this paper.
